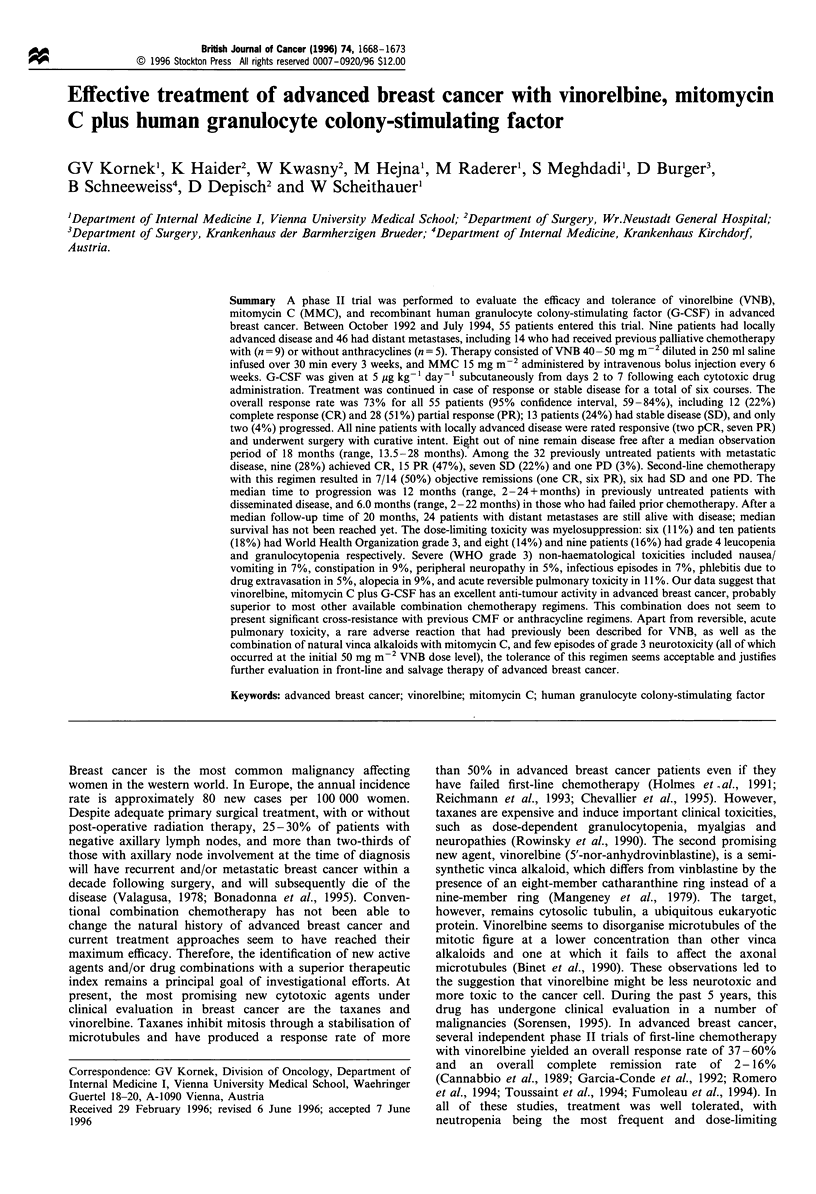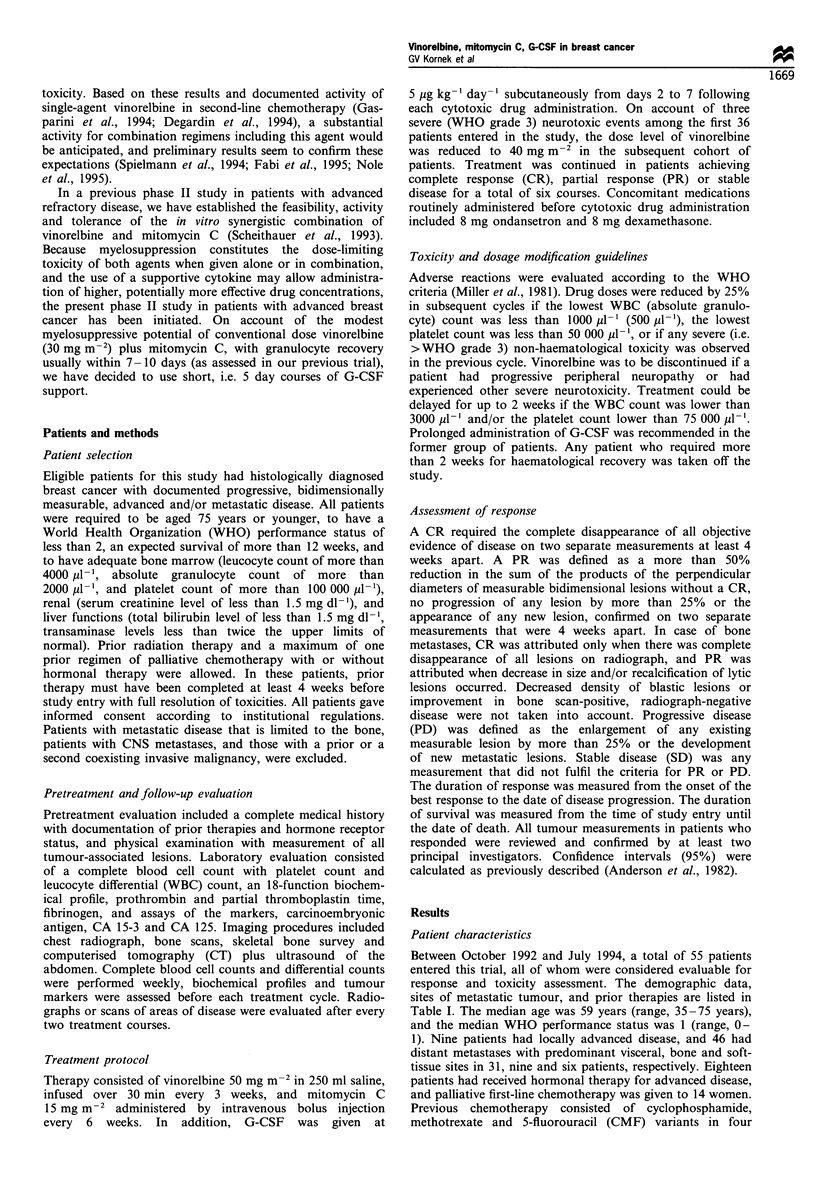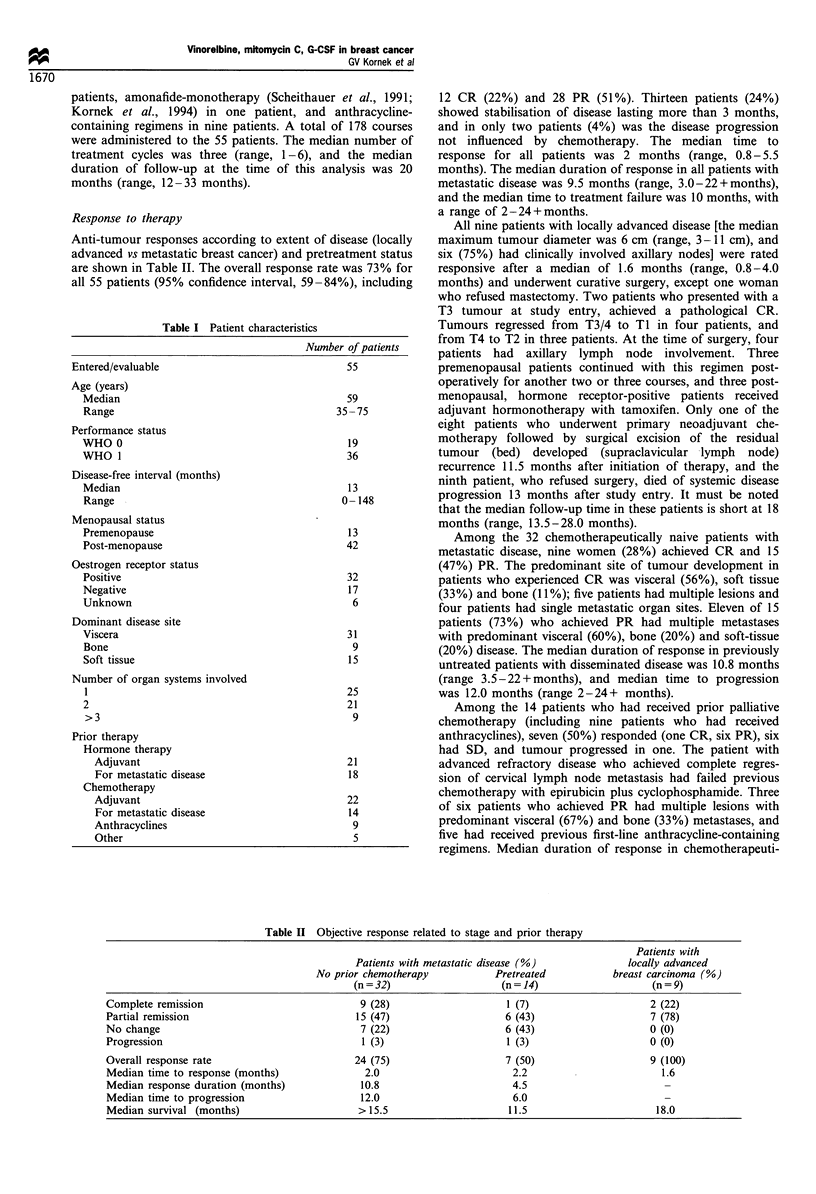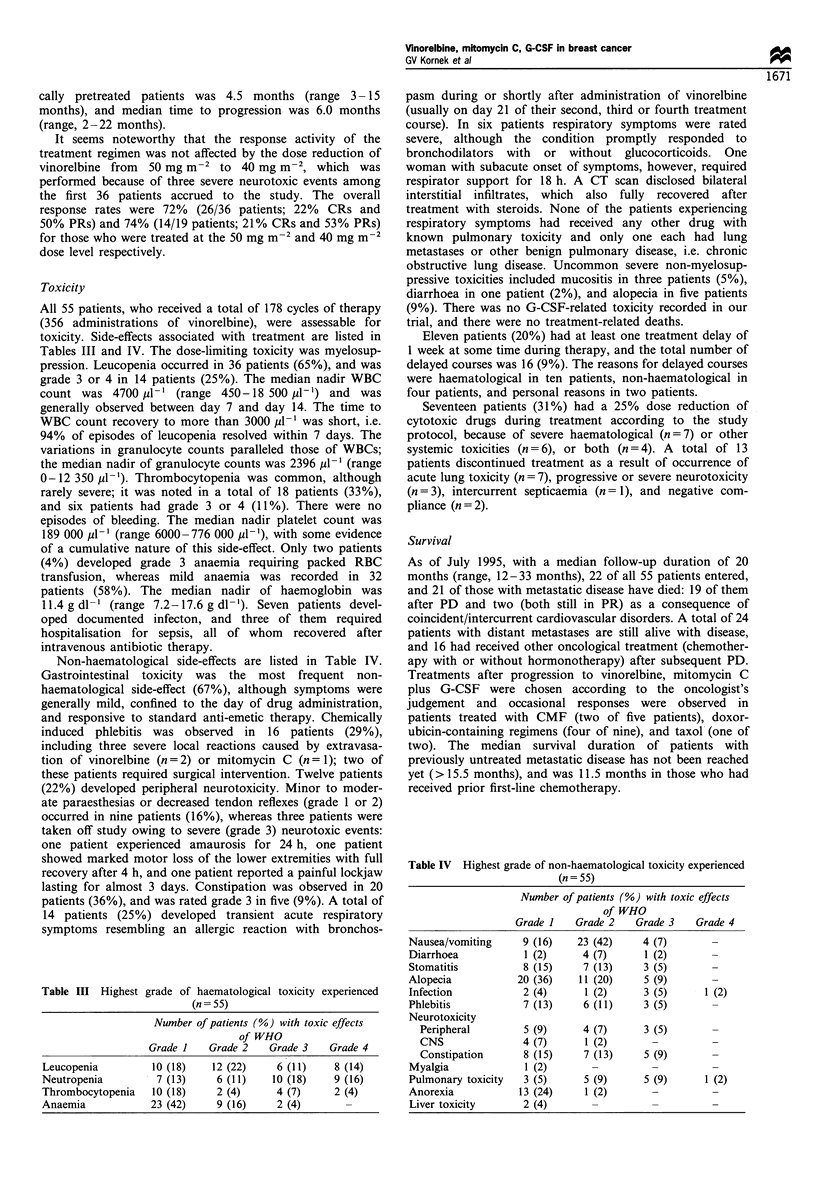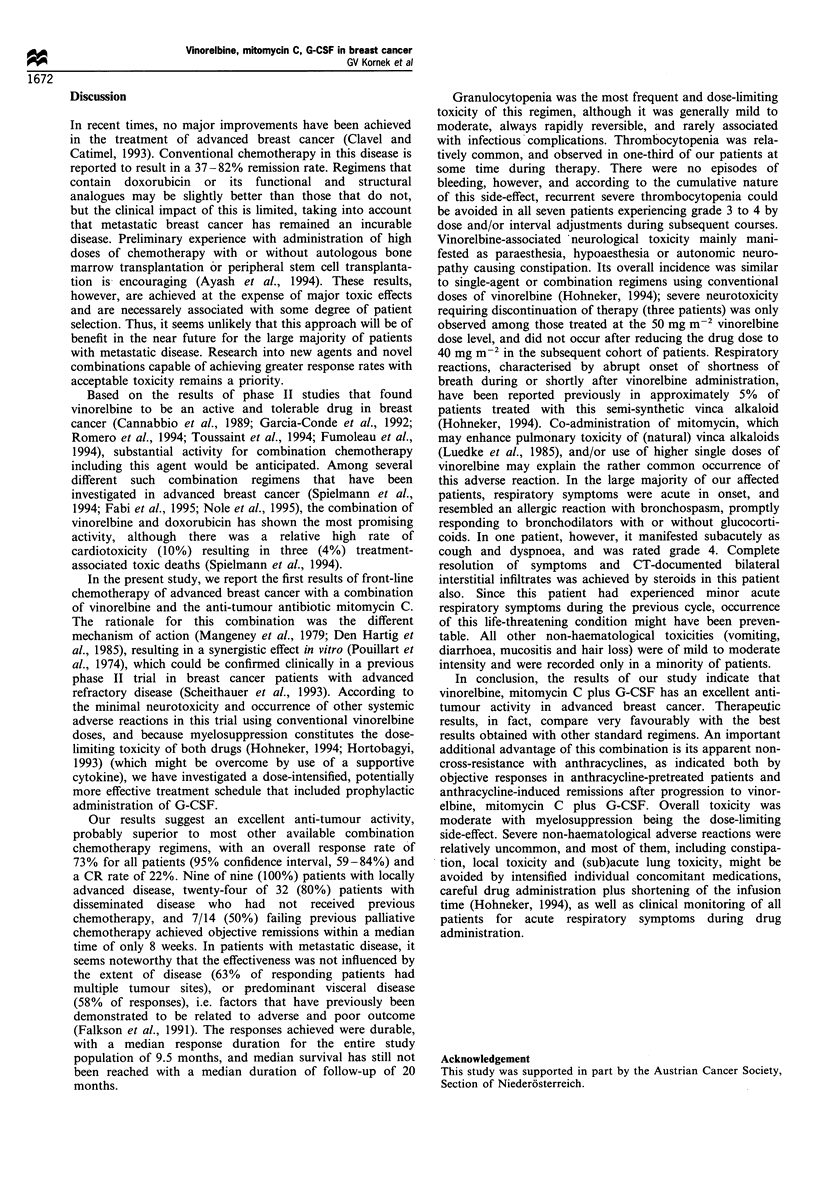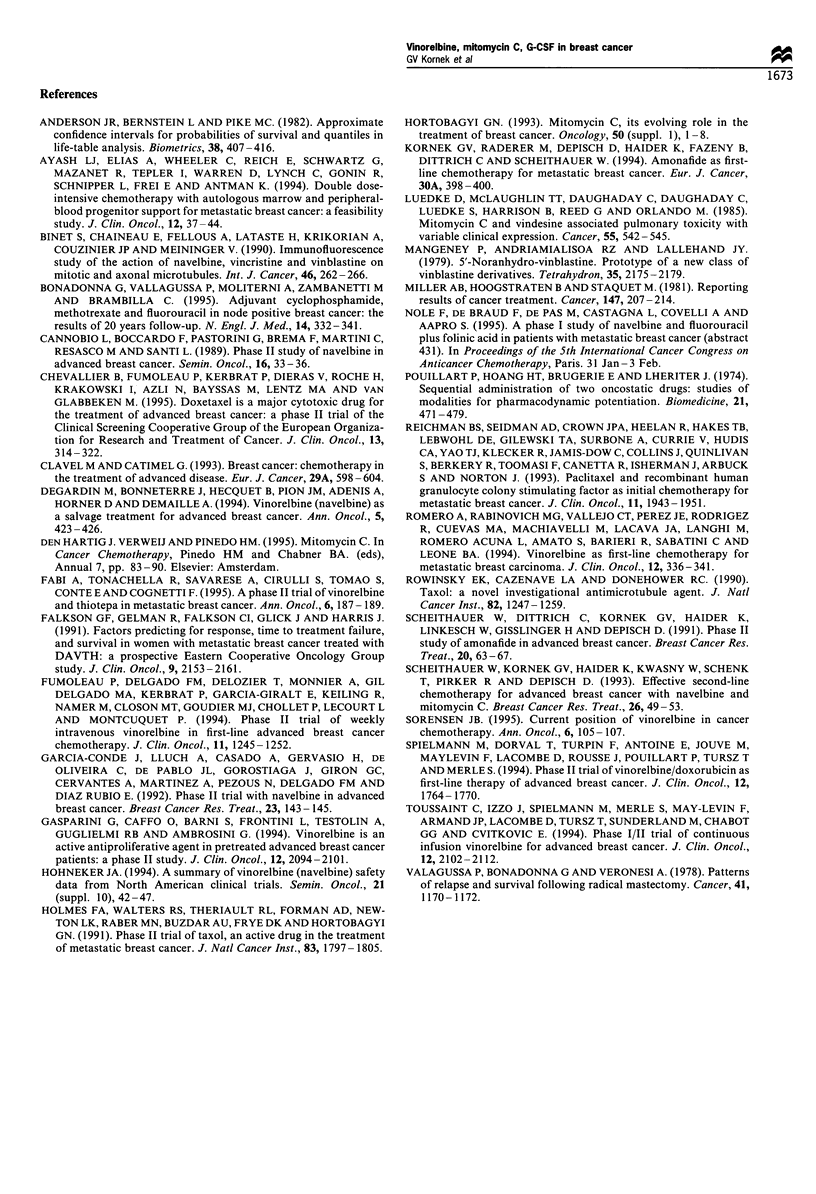# Effective treatment of advanced breast cancer with vinorelbine, mitomycin C plus human granulocyte colony-stimulating factor.

**DOI:** 10.1038/bjc.1996.607

**Published:** 1996-11

**Authors:** G. V. Kornek, K. Haider, W. Kwasny, M. Hejna, M. Raderer, S. Meghdadi, D. Burger, B. Schneeweiss, D. Depisch, W. Scheithauer

**Affiliations:** Department of Internal Medicine I, Vienna University Medical School, Austria.

## Abstract

A phase II trial was performed to evaluate the efficacy and tolerance of vinorelbine (VNB), mitomycin C (MMC), and recombinant human granulocyte colony-stimulating factor (G-CSF) in advanced breast cancer. Between October 1992 and July 1994, 55 patients entered this trial. Nine patients had locally advanced disease and 46 had distant metastases, including 14 who had received previous palliative chemotherapy with (n = 9) or without anthracyclines (n = 5). Therapy consisted of VNB 40-50 mg m(-2) diluted in 250 ml saline infused over 30 min every 3 weeks, and MMC 15 mg m(-2) administered by intravenous bolus injection every 6 weeks. G-CSF was given at 5 microg kg(-1) day(-1) subcutaneously from days 2 to 7 following each cytotoxic drug administration. Treatment was continued in case of response or stable disease for a total of six courses. The overall response rate was 73% for all 55 patients (95% confidence interval, 59-84%), including 12 (22%) complete response (CR) and 28 (51%) partial response (PR); 13 patients (24%) had stable disease (SD), and only two (4%) progressed. All nine patients with locally advanced disease were rated responsive (two pCR, seven PR) and underwent surgery with curative intent. Eight out of nine remain disease free after a median observation period of 18 months (range, 13.5-28 months). Among the 32 previously untreated patients with metastatic disease, nine (28%) achieved CR, 15 PR (47%), seven SD (22%) and one PD (3%). Second-line chemotherapy with this regimen resulted in 7/14 (50%) objective remissions (one CR, six PR), six had SD and one PD. The median time to progression was 12 months (range, 2-24+ months) in previously untreated patients with disseminated disease, and 6.0 months (range, 2-22 months) in those who had failed prior chemotherapy. After a median follow-up time of 20 months, 24 patients with distant metastases are still alive with disease; median survival has not been reached yet. The dose-limiting toxicity was myelosuppression: six (11%) and ten patients (18%) had World Health Organization grade 3, and eight (14%) and nine patients (16%) had grade 4 leucopenia and granulocytopenia respectively. Severe (WHO grade 3) non-haematological toxicities included nausea/vomiting in 7%, constipation in 9%, peripheral neuropathy in 5%, infectious episodes in 7%, phlebitis due to drug extravasation in 5%, alopecia in 9%, and acute reversible pulmonary toxicity in 11%. Our data suggest that vinorelbine, mitomycin C plus G-CSF has an excellent anti-tumour activity in advanced breast cancer, probably superior to most other available combination chemotherapy regimens. This combination does not seem to present significant cross-resistance with previous CMF or anthracycline regimens. Apart from reversible, acute pulmonary toxicity, a rare adverse reaction that had previously been described for VNB, as well as the combination of natural vinca alkaloids with mitomycin C, and few episodes of grade 3 neurotoxicity (all of which occurred at the initial 50 mg m(-2) VNB dose level), the tolerance of this regimen seems acceptable and justifies further evaluation in front-line and salvage therapy of advanced breast cancer.


British Journal of Cancer (1996) 74, 1668-1673
© 1996 Stockton Press All rights reserved 0007-0920/96 $12.00

### Effective treatment of advanced breast cancer with vinorelbine, mitomycin C plus human granulocyte colony-stimulating factor

GV Kornek[1], K Haider[2], W Kwasny[2], M Hejna[1], M Raderer[1], S Meghdadi[1], D Burger[3], B Schneeweiss[4], D Depisch[2] and W Scheithauer[1]

[1]*Department of Internal Medicine I, Vienna University Medical School;* [2]*Department of Surgery, Wr.Neustadt General Hospital;* [3]*Department of Surgery, Krankenhaus der Barmherzigen Brueder;* [4]*Department of Internal Medicine, Krankenhaus Kirchdorf, Austria.*

**Summary** A phase II trial was performed to evaluate the efficacy and tolerance of vinorelbine (VNB), mitomycin C (MMC), and recombinant human granulocyte colony-stimulating factor (G-CSF) in advanced breast cancer. Between October 1992 and July 1994, 55 patients entered this trial. Nine patients had locally advanced disease and 46 had distant metastases, including 14 who had received previous palliative chemotherapy with ($n=9$) or without anthracyclines ($n=5$). Therapy consisted of VNB 40-50 mg $m^{-2}$ diluted in 250 ml saline infused over 30 min every 3 weeks, and MMC 15 mg $m^{-2}$ administered by intravenous bolus injection every 6 weeks. G-CSF was given at 5 $\mu g\ kg^{-1}\ day^{-1}$ subcutaneously from days 2 to 7 following each cytotoxic drug administration. Treatment was continued in case of response or stable disease for a total of six courses. The overall response rate was 73% for all 55 patients (95% confidence interval, 59-84%), including 12 (22%) complete response (CR) and 28 (51%) partial response (PR); 13 patients (24%) had stable disease (SD), and only two (4%) progressed. All nine patients with locally advanced disease were rated responsive (two pCR, seven PR) and underwent surgery with curative intent. Eight out of nine remain disease free after a median observation period of 18 months (range, 13.5-28 months). Among the 32 previously untreated patients with metastatic disease, nine (28%) achieved CR, 15 PR (47%), seven SD (22%) and one PD (3%). Second-line chemotherapy with this regimen resulted in 7/14 (50%) objective remissions (one CR, six PR), six had SD and one PD. The median time to progression was 12 months (range, 2-24+months) in previously untreated patients with disseminated disease, and 6.0 months (range, 2-22 months) in those who had failed prior chemotherapy. After a median follow-up time of 20 months, 24 patients with distant metastases are still alive with disease; median survival has not been reached yet. The dose-limiting toxicity was myelosuppression: six (11%) and ten patients (18%) had World Health Organization grade 3, and eight (14%) and nine patients (16%) had grade 4 leucopenia and granulocytopenia respectively. Severe (WHO grade 3) non-haematological toxicities included nausea/vomiting in 7%, constipation in 9%, peripheral neuropathy in 5%, infectious episodes in 7%, phlebitis due to drug extravasation in 5%, alopecia in 9%, and acute reversible pulmonary toxicity in 11%. Our data suggest that vinorelbine, mitomycin C plus G-CSF has an excellent anti-tumour activity in advanced breast cancer, probably superior to most other available combination chemotherapy regimens. This combination does not seem to present significant cross-resistance with previous CMF or anthracycline regimens. Apart from reversible, acute pulmonary toxicity, a rare adverse reaction that had previously been described for VNB, as well as the combination of natural vinca alkaloids with mitomycin C, and few episodes of grade 3 neurotoxicity (all of which occurred at the initial 50 mg $m^{-2}$ VNB dose level), the tolerance of this regimen seems acceptable and justifies further evaluation in front-line and salvage therapy of advanced breast cancer.

**Keywords:** advanced breast cancer; vinorelbine; mitomycin C; human granulocyte colony-stimulating factor

Breast cancer is the most common malignancy affecting women in the western world. In Europe, the annual incidence rate is approximately 80 new cases per 100 000 women. Despite adequate primary surgical treatment, with or without post-operative radiation therapy, 25-30% of patients with negative axillary lymph nodes, and more than two-thirds of those with axillary node involvement at the time of diagnosis will have recurrent and/or metastatic breast cancer within a decade following surgery, and will subsequently die of the disease (Valagusa, 1978; Bonadonna *et al.*, 1995). Conventional combination chemotherapy has not been able to change the natural history of advanced breast cancer and current treatment approaches seem to have reached their maximum efficacy. Therefore, the identification of new active agents and/or drug combinations with a superior therapeutic index remains a principal goal of investigational efforts. At present, the most promising new cytotoxic agents under clinical evaluation in breast cancer are the taxanes and vinorelbine. Taxanes inhibit mitosis through a stabilisation of microtubules and have produced a response rate of more than 50% in advanced breast cancer patients even if they have failed first-line chemotherapy (Holmes *et al.*, 1991; Reichmann *et al.*, 1993; Chevallier *et al.*, 1995). However, taxanes are expensive and induce important clinical toxicities, such as dose-dependent granulocytopenia, myalgias and neuropathies (Rowinsky *et al.*, 1990). The second promising new agent, vinorelbine (5′-nor-anhydrovinblastine), is a semi-synthetic vinca alkaloid, which differs from vinblastine by the presence of an eight-member catharanthine ring instead of a nine-member ring (Mangeney *et al.*, 1979). The target, however, remains cytosolic tubulin, a ubiquitous eukaryotic protein. Vinorelbine seems to disorganise microtubules of the mitotic figure at a lower concentration than other vinca alkaloids and one at which it fails to affect the axonal microtubules (Binet *et al.*, 1990). These observations led to the suggestion that vinorelbine might be less neurotoxic and more toxic to the cancer cell. During the past 5 years, this drug has undergone clinical evaluation in a number of malignancies (Sorensen, 1995). In advanced breast cancer, several independent phase II trials of first-line chemotherapy with vinorelbine yielded an overall response rate of 37-60% and an overall complete remission rate of 2-16% (Cannabbio *et al.*, 1989; Garcia-Conde *et al.*, 1992; Romero *et al.*, 1994; Toussaint *et al.*, 1994; Fumoleau *et al.*, 1994). In all of these studies, treatment was well tolerated, with neutropenia being the most frequent and dose-limiting

Correspondence: GV Kornek, Division of Oncology, Department of Internal Medicine I, Vienna University Medical School, Waehringer Guertel 18-20, A-1090 Vienna, Austria
Received 29 February 1996; revised 6 June 1996; accepted 7 June 1996

toxicity. Based on these results and documented activity of single-agent vinorelbine in second-line chemotherapy (Gasparini *et al.*, 1994; Degardin *et al.*, 1994), a substantial activity for combination regimens including this agent would be anticipated, and preliminary results seem to confirm these expectations (Spielmann *et al.*, 1994; Fabi *et al.*, 1995; Nole *et al.*, 1995).

In a previous phase II study in patients with advanced refractory disease, we have established the feasibility, activity and tolerance of the *in vitro* synergistic combination of vinorelbine and mitomycin C (Scheithauer *et al.*, 1993). Because myelosuppression constitutes the dose-limiting toxicity of both agents when given alone or in combination, and the use of a supportive cytokine may allow administration of higher, potentially more effective drug concentrations, the present phase II study in patients with advanced breast cancer has been initiated. On account of the modest myelosuppressive potential of conventional dose vinorelbine (30 mg $m^{-2}$) plus mitomycin C, with granulocyte recovery usually within 7–10 days (as assessed in our previous trial), we have decided to use short, i.e. 5 day courses of G-CSF support.

### Patients and methods

### *Patient selection*

Eligible patients for this study had histologically diagnosed breast cancer with documented progressive, bidimensionally measurable, advanced and/or metastatic disease. All patients were required to be aged 75 years or younger, to have a World Health Organization (WHO) performance status of less than 2, an expected survival of more than 12 weeks, and to have adequate bone marrow (leucocyte count of more than 4000 $\mu l^{-1}$, absolute granulocyte count of more than 2000 $\mu l^{-1}$, and platelet count of more than 100 000 $\mu l^{-1}$), renal (serum creatinine level of less than 1.5 mg $dl^{-1}$), and liver functions (total bilirubin level of less than 1.5 mg $dl^{-1}$, transaminase levels less than twice the upper limits of normal). Prior radiation therapy and a maximum of one prior regimen of palliative chemotherapy with or without hormonal therapy were allowed. In these patients, prior therapy must have been completed at least 4 weeks before study entry with full resolution of toxicities. All patients gave informed consent according to institutional regulations. Patients with metastatic disease that is limited to the bone, patients with CNS metastases, and those with a prior or a second coexisting invasive malignancy, were excluded.

### *Pretreatment and follow-up evaluation*

Pretreatment evaluation included a complete medical history with documentation of prior therapies and hormone receptor status, and physical examination with measurement of all tumour-associated lesions. Laboratory evaluation consisted of a complete blood cell count with platelet count and leucocyte differential (WBC) count, an 18-function biochemical profile, prothrombin and partial thromboplastin time, fibrinogen, and assays of the markers, carcinoembryonic antigen, CA 15-3 and CA 125. Imaging procedures included chest radiograph, bone scans, skeletal bone survey and computerised tomography (CT) plus ultrasound of the abdomen. Complete blood cell counts and differential counts were performed weekly, biochemical profiles and tumour markers were assessed before each treatment cycle. Radiographs or scans of areas of disease were evaluated after every two treatment courses.

### *Treatment protocol*

Therapy consisted of vinorelbine 50 mg $m^{-2}$ in 250 ml saline, infused over 30 min every 3 weeks, and mitomycin C 15 mg $m^{-2}$ administered by intravenous bolus injection every 6 weeks. In addition, G-CSF was given at 5 $\mu g$ $kg^{-1}$ $day^{-1}$ subcutaneously from days 2 to 7 following each cytotoxic drug administration. On account of three severe (WHO grade 3) neurotoxic events among the first 36 patients entered in the study, the dose level of vinorelbine was reduced to 40 mg $m^{-2}$ in the subsequent cohort of patients. Treatment was continued in patients achieving complete response (CR), partial response (PR) or stable disease for a total of six courses. Concomitant medications routinely administered before cytotoxic drug administration included 8 mg ondansetron and 8 mg dexamethasone.

### *Toxicity and dosage modification guidelines*

Adverse reactions were evaluated according to the WHO criteria (Miller *et al.*, 1981). Drug doses were reduced by 25% in subsequent cycles if the lowest WBC (absolute granulocyte) count was less than 1000 $\mu l^{-1}$ (500 $\mu l^{-1}$), the lowest platelet count was less than 50 000 $\mu l^{-1}$, or if any severe (i.e. >WHO grade 3) non-haematological toxicity was observed in the previous cycle. Vinorelbine was to be discontinued if a patient had progressive peripheral neuropathy or had experienced other severe neurotoxicity. Treatment could be delayed for up to 2 weeks if the WBC count was lower than 3000 $\mu l^{-1}$ and/or the platelet count lower than 75 000 $\mu l^{-1}$. Prolonged administration of G-CSF was recommended in the former group of patients. Any patient who required more than 2 weeks for haematological recovery was taken off the study.

### *Assessment of response*

A CR required the complete disappearance of all objective evidence of disease on two separate measurements at least 4 weeks apart. A PR was defined as a more than 50% reduction in the sum of the products of the perpendicular diameters of measurable bidimensional lesions without a CR, no progression of any lesion by more than 25% or the appearance of any new lesion, confirmed on two separate measurements that were 4 weeks apart. In case of bone metastases, CR was attributed only when there was complete disappearance of all lesions on radiograph, and PR was attributed when decrease in size and/or recalcification of lytic lesions occurred. Decreased density of blastic lesions or improvement in bone scan-positive, radiograph-negative disease were not taken into account. Progressive disease (PD) was defined as the enlargement of any existing measurable lesion by more than 25% or the development of new metastatic lesions. Stable disease (SD) was any measurement that did not fulfil the criteria for PR or PD. The duration of response was measured from the onset of the best response to the date of disease progression. The duration of survival was measured from the time of study entry until the date of death. All tumour measurements in patients who responded were reviewed and confirmed by at least two principal investigators. Confidence intervals (95%) were calculated as previously described (Anderson *et al.*, 1982).

### Results

### *Patient characteristics*

Between October 1992 and July 1994, a total of 55 patients entered this trial, all of whom were considered evaluable for response and toxicity assessment. The demographic data, sites of metastatic tumour, and prior therapies are listed in Table I. The median age was 59 years (range, 35–75 years), and the median WHO performance status was 1 (range, 0–1). Nine patients had locally advanced disease, and 46 had distant metastases with predominant visceral, bone and soft-tissue sites in 31, nine and six patients, respectively. Eighteen patients had received hormonal therapy for advanced disease, and palliative first-line chemotherapy was given to 14 women. Previous chemotherapy consisted of cyclophosphamide, methotrexate and 5-fluorouracil (CMF) variants in four patients, amonafide-monotherapy (Scheithauer *et al.*, 1991; Kornek *et al.*, 1994) in one patient, and anthracycline-containing regimens in nine patients. A total of 178 courses were administered to the 55 patients. The median number of treatment cycles was three (range, 1–6), and the median duration of follow-up at the time of this analysis was 20 months (range, 12–33 months).

*Response to therapy*

Anti-tumour responses according to extent of disease (locally advanced *vs* metastatic breast cancer) and pretreatment status are shown in Table II. The overall response rate was 73% for all 55 patients (95% confidence interval, 59–84%), including 12 CR (22%) and 28 PR (51%). Thirteen patients (24%) showed stabilisation of disease lasting more than 3 months, and in only two patients (4%) was the disease progression not influenced by chemotherapy. The median time to response for all patients was 2 months (range, 0.8–5.5 months). The median duration of response in all patients with metastatic disease was 9.5 months (range, 3.0–22+months), and the median time to treatment failure was 10 months, with a range of 2–24+months.

All nine patients with locally advanced disease [the median maximum tumour diameter was 6 cm (range, 3–11 cm), and six (75%) had clinically involved axillary nodes] were rated responsive after a median of 1.6 months (range, 0.8–4.0 months) and underwent curative surgery, except one woman who refused mastectomy. Two patients who presented with a T3 tumour at study entry, achieved a pathological CR. Tumours regressed from T3/4 to T1 in four patients, and from T4 to T2 in three patients. At the time of surgery, four patients had axillary lymph node involvement. Three premenopausal patients continued with this regimen post-operatively for another two or three courses, and three post-menopausal, hormone receptor-positive patients received adjuvant hormonotherapy with tamoxifen. Only one of the eight patients who underwent primary neoadjuvant chemotherapy followed by surgical excision of the residual tumour (bed) developed (supraclavicular lymph node) recurrence 11.5 months after initiation of therapy, and the ninth patient, who refused surgery, died of systemic disease progression 13 months after study entry. It must be noted that the median follow-up time in these patients is short at 18 months (range, 13.5–28.0 months).

Among the 32 chemotherapeutically naive patients with metastatic disease, nine women (28%) achieved CR and 15 (47%) PR. The predominant site of tumour development in patients who experienced CR was visceral (56%), soft tissue (33%) and bone (11%); five patients had multiple lesions and four patients had single metastatic organ sites. Eleven of 15 patients (73%) who achieved PR had multiple metastases with predominant visceral (60%), bone (20%) and soft-tissue (20%) disease. The median duration of response in previously untreated patients with disseminated disease was 10.8 months (range 3.5–22+months), and median time to progression was 12.0 months (range 2–24+ months).

Among the 14 patients who had received prior palliative chemotherapy (including nine patients who had received anthracyclines), seven (50%) responded (one CR, six PR), six had SD, and tumour progressed in one. The patient with advanced refractory disease who achieved complete regression of cervical lymph node metastasis had failed previous chemotherapy with epirubicin plus cyclophosphamide. Three of six patients who achieved PR had multiple lesions with predominant visceral (67%) and bone (33%) metastases, and five had received previous first-line anthracycline-containing regimens. Median duration of response in chemotherapeuti-

**Table I** Patient characteristics

| | *Number of patients* |
|---|---|
| Entered/evaluable | 55 |
| Age (years) | |
| Median | 59 |
| Range | 35–75 |
| Performance status | |
| WHO 0 | 19 |
| WHO 1 | 36 |
| Disease-free interval (months) | |
| Median | 13 |
| Range | 0–148 |
| Menopausal status | |
| Premenopause | 13 |
| Post-menopause | 42 |
| Oestrogen receptor status | |
| Positive | 32 |
| Negative | 17 |
| Unknown | 6 |
| Dominant disease site | |
| Viscera | 31 |
| Bone | 9 |
| Soft tissue | 15 |
| Number of organ systems involved | |
| 1 | 25 |
| 2 | 21 |
| >3 | 9 |
| Prior therapy | |
| Hormone therapy | |
| Adjuvant | 21 |
| For metastatic disease | 18 |
| Chemotherapy | |
| Adjuvant | 22 |
| For metastatic disease | 14 |
| Anthracyclines | 9 |
| Other | 5 |

**Table II** Objective response related to stage and prior therapy

| | *Patients with metastatic disease (%)* No prior chemotherapy (n=*32*) | Pretreated (n=*14*) | *Patients with locally advanced breast carcinoma (%)* (n=*9*) |
|---|---|---|---|
| Complete remission | 9 (28) | 1 (7) | 2 (22) |
| Partial remission | 15 (47) | 6 (43) | 7 (78) |
| No change | 7 (22) | 6 (43) | 0 (0) |
| Progression | 1 (3) | 1 (3) | 0 (0) |
| Overall response rate | 24 (75) | 7 (50) | 9 (100) |
| Median time to response (months) | 2.0 | 2.2 | 1.6 |
| Median response duration (months) | 10.8 | 4.5 | – |
| Median time to progression | 12.0 | 6.0 | – |
| Median survival (months) | >15.5 | 11.5 | 18.0 |

cally pretreated patients was 4.5 months (range 3–15 months), and median time to progression was 6.0 months (range, 2–22 months).

It seems noteworthy that the response activity of the treatment regimen was not affected by the dose reduction of vinorelbine from 50 mg $m^{-2}$ to 40 mg $m^{-2}$, which was performed because of three severe neurotoxic events among the first 36 patients accrued to the study. The overall response rates were 72% (26/36 patients; 22% CRs and 50% PRs) and 74% (14/19 patients; 21% CRs and 53% PRs) for those who were treated at the 50 mg $m^{-2}$ and 40 mg $m^{-2}$ dose level respectively.

*Toxicity*

All 55 patients, who received a total of 178 cycles of therapy (356 administrations of vinorelbine), were assessable for toxicity. Side-effects associated with treatment are listed in Tables III and IV. The dose-limiting toxicity was myelosuppression. Leucopenia occurred in 36 patients (65%), and was grade 3 or 4 in 14 patients (25%). The median nadir WBC count was 4700 $\mu l^{-1}$ (range 450–18 500 $\mu l^{-1}$) and was generally observed between day 7 and day 14. The time to WBC count recovery to more than 3000 $\mu l^{-1}$ was short, i.e. 94% of episodes of leucopenia resolved within 7 days. The variations in granulocyte counts paralleled those of WBCs; the median nadir of granulocyte counts was 2396 $\mu l^{-1}$ (range 0–12 350 $\mu l^{-1}$). Thrombocytopenia was common, although rarely severe; it was noted in a total of 18 patients (33%), and six patients had grade 3 or 4 (11%). There were no episodes of bleeding. The median nadir platelet count was 189 000 $\mu l^{-1}$ (range 6000–776 000 $\mu l^{-1}$), with some evidence of a cumulative nature of this side-effect. Only two patients (4%) developed grade 3 anaemia requiring packed RBC transfusion, whereas mild anaemia was recorded in 32 patients (58%). The median nadir of haemoglobin was 11.4 g $dl^{-1}$ (range 7.2–17.6 g $dl^{-1}$). Seven patients developed documented infecton, and three of them required hospitalisation for sepsis, all of whom recovered after intravenous antibiotic therapy.

Non-haematological side-effects are listed in Table IV. Gastrointestinal toxicity was the most frequent non-haematological side-effect (67%), although symptoms were generally mild, confined to the day of drug administration, and responsive to standard anti-emetic therapy. Chemically induced phlebitis was observed in 16 patients (29%), including three severe local reactions caused by extravasation of vinorelbine ($n=2$) or mitomycin C ($n=1$); two of these patients required surgical intervention. Twelve patients (22%) developed peripheral neurotoxicity. Minor to moderate paraesthesias or decreased tendon reflexes (grade 1 or 2) occurred in nine patients (16%), whereas three patients were taken off study owing to severe (grade 3) neurotoxic events: one patient experienced amaurosis for 24 h, one patient showed marked motor loss of the lower extremities with full recovery after 4 h, and one patient reported a painful lockjaw lasting for almost 3 days. Constipation was observed in 20 patients (36%), and was rated grade 3 in five (9%). A total of 14 patients (25%) developed transient acute respiratory symptoms resembling an allergic reaction with bronchospasm during or shortly after administration of vinorelbine (usually on day 21 of their second, third or fourth treatment course). In six patients respiratory symptoms were rated severe, although the condition promptly responded to bronchodilators with or without glucocorticoids. One woman with subacute onset of symptoms, however, required respirator support for 18 h. A CT scan disclosed bilateral interstitial infiltrates, which also fully recovered after treatment with steroids. None of the patients experiencing respiratory symptoms had received any other drug with known pulmonary toxicity and only one each had lung metastases or other benign pulmonary disease, i.e. chronic obstructive lung disease. Uncommon severe non-myelosuppressive toxicities included mucositis in three patients (5%), diarrhoea in one patient (2%), and alopecia in five patients (9%). There was no G-CSF-related toxicity recorded in our trial, and there were no treatment-related deaths.

Eleven patients (20%) had at least one treatment delay of 1 week at some time during therapy, and the total number of delayed courses was 16 (9%). The reasons for delayed courses were haematological in ten patients, non-haematological in four patients, and personal reasons in two patients.

Seventeen patients (31%) had a 25% dose reduction of cytotoxic drugs during treatment according to the study protocol, because of severe haematological ($n=7$) or other systemic toxicities ($n=6$), or both ($n=4$). A total of 13 patients discontinued treatment as a result of occurrence of acute lung toxicity ($n=7$), progressive or severe neurotoxicity ($n=3$), intercurrent septicaemia ($n=1$), and negative compliance ($n=2$).

*Survival*

As of July 1995, with a median follow-up duration of 20 months (range, 12–33 months), 22 of all 55 patients entered, and 21 of those with metastatic disease have died: 19 of them after PD and two (both still in PR) as a consequence of coincident/intercurrent cardiovascular disorders. A total of 24 patients with distant metastases are still alive with disease, and 16 had received other oncological treatment (chemotherapy with or without hormonotherapy) after subsequent PD. Treatments after progression to vinorelbine, mitomycin C plus G-CSF were chosen according to the oncologist's judgement and occasional responses were observed in patients treated with CMF (two of five patients), doxorubicin-containing regimens (four of nine), and taxol (one of two). The median survival duration of patients with previously untreated metastatic disease has not been reached yet (>15.5 months), and was 11.5 months in those who had received prior first-line chemotherapy.

**Table III** Highest grade of haematological toxicity experienced ($n=55$)

| | *Number of patients (%) with toxic effects of WHO* | | | |
|---|---|---|---|---|
| | *Grade 1* | *Grade 2* | *Grade 3* | *Grade 4* |
| Leucopenia | 10 (18) | 12 (22) | 6 (11) | 8 (14) |
| Neutropenia | 7 (13) | 6 (11) | 10 (18) | 9 (16) |
| Thrombocytopenia | 10 (18) | 2 (4) | 4 (7) | 2 (4) |
| Anaemia | 23 (42) | 9 (16) | 2 (4) | – |

**Table IV** Highest grade of non-haematological toxicity experienced ($n=55$)

| | *Number of patients (%) with toxic effects of WHO* | | | |
|---|---|---|---|---|
| | *Grade 1* | *Grade 2* | *Grade 3* | *Grade 4* |
| Nausea/vomiting | 9 (16) | 23 (42) | 4 (7) | – |
| Diarrhoea | 1 (2) | 4 (7) | 1 (2) | – |
| Stomatitis | 8 (15) | 7 (13) | 3 (5) | – |
| Alopecia | 20 (36) | 11 (20) | 5 (9) | – |
| Infection | 2 (4) | 1 (2) | 3 (5) | 1 (2) |
| Phlebitis | 7 (13) | 6 (11) | 3 (5) | – |
| Neurotoxicity | | | | |
| Peripheral | 5 (9) | 4 (7) | 3 (5) | – |
| CNS | 4 (7) | 1 (2) | – | – |
| Constipation | 8 (15) | 7 (13) | 5 (9) | – |
| Myalgia | 1 (2) | – | – | – |
| Pulmonary toxicity | 3 (5) | 5 (9) | 5 (9) | 1 (2) |
| Anorexia | 13 (24) | 1 (2) | – | – |
| Liver toxicity | 2 (4) | – | – | – |

### Discussion

In recent times, no major improvements have been achieved in the treatment of advanced breast cancer (Clavel and Catimel, 1993). Conventional chemotherapy in this disease is reported to result in a 37–82% remission rate. Regimens that contain doxorubicin or its functional and structural analogues may be slightly better than those that do not, but the clinical impact of this is limited, taking into account that metastatic breast cancer has remained an incurable disease. Preliminary experience with administration of high doses of chemotherapy with or without autologous bone marrow transplantation or peripheral stem cell transplantation is encouraging (Ayash *et al.*, 1994). These results, however, are achieved at the expense of major toxic effects and are necessarely associated with some degree of patient selection. Thus, it seems unlikely that this approach will be of benefit in the near future for the large majority of patients with metastatic disease. Research into new agents and novel combinations capable of achieving greater response rates with acceptable toxicity remains a priority.

Based on the results of phase II studies that found vinorelbine to be an active and tolerable drug in breast cancer (Cannabbio *et al.*, 1989; Garcia-Conde *et al.*, 1992; Romero *et al.*, 1994; Toussaint *et al.*, 1994; Fumoleau *et al.*, 1994), substantial activity for combination chemotherapy including this agent would be anticipated. Among several different such combination regimens that have been investigated in advanced breast cancer (Spielmann *et al.*, 1994; Fabi *et al.*, 1995; Nole *et al.*, 1995), the combination of vinorelbine and doxorubicin has shown the most promising activity, although there was a relative high rate of cardiotoxicity (10%) resulting in three (4%) treatment-associated toxic deaths (Spielmann *et al.*, 1994).

In the present study, we report the first results of front-line chemotherapy of advanced breast cancer with a combination of vinorelbine and the anti-tumour antibiotic mitomycin C. The rationale for this combination was the different mechanism of action (Mangeney *et al.*, 1979; Den Hartig *et al.*, 1985), resulting in a synergistic effect *in vitro* (Pouillart *et al.*, 1974), which could be confirmed clinically in a previous phase II trial in breast cancer patients with advanced refractory disease (Scheithauer *et al.*, 1993). According to the minimal neurotoxicity and occurrence of other systemic adverse reactions in this trial using conventional vinorelbine doses, and because myelosuppression constitutes the dose-limiting toxicity of both drugs (Hohneker, 1994; Hortobagyi, 1993) (which might be overcome by use of a supportive cytokine), we have investigated a dose-intensified, potentially more effective treatment schedule that included prophylactic administration of G-CSF.

Our results suggest an excellent anti-tumour activity, probably superior to most other available combination chemotherapy regimens, with an overall response rate of 73% for all patients (95% confidence interval, 59–84%) and a CR rate of 22%. Nine of nine (100%) patients with locally advanced disease, twenty-four of 32 (80%) patients with disseminated disease who had not received previous chemotherapy, and 7/14 (50%) failing previous palliative chemotherapy achieved objective remissions within a median time of only 8 weeks. In patients with metastatic disease, it seems noteworthy that the effectiveness was not influenced by the extent of disease (63% of responding patients had multiple tumour sites), or predominant visceral disease (58% of responses), i.e. factors that have previously been demonstrated to be related to adverse and poor outcome (Falkson *et al.*, 1991). The responses achieved were durable, with a median response duration for the entire study population of 9.5 months, and median survival has still not been reached with a median duration of follow-up of 20 months.

Granulocytopenia was the most frequent and dose-limiting toxicity of this regimen, although it was generally mild to moderate, always rapidly reversible, and rarely associated with infectious complications. Thrombocytopenia was relatively common, and observed in one-third of our patients at some time during therapy. There were no episodes of bleeding, however, and according to the cumulative nature of this side-effect, recurrent severe thrombocytopenia could be avoided in all seven patients experiencing grade 3 to 4 by dose and/or interval adjustments during subsequent courses. Vinorelbine-associated neurological toxicity mainly manifested as paraesthesia, hypoaesthesia or autonomic neuropathy causing constipation. Its overall incidence was similar to single-agent or combination regimens using conventional doses of vinorelbine (Hohneker, 1994); severe neurotoxicity requiring discontinuation of therapy (three patients) was only observed among those treated at the 50 mg $m^{-2}$ vinorelbine dose level, and did not occur after reducing the drug dose to 40 mg $m^{-2}$ in the subsequent cohort of patients. Respiratory reactions, characterised by abrupt onset of shortness of breath during or shortly after vinorelbine administration, have been reported previously in approximately 5% of patients treated with this semi-synthetic vinca alkaloid (Hohneker, 1994). Co-administration of mitomycin, which may enhance pulmonary toxicity of (natural) vinca alkaloids (Luedke *et al.*, 1985), and/or use of higher single doses of vinorelbine may explain the rather common occurrence of this adverse reaction. In the large majority of our affected patients, respiratory symptoms were acute in onset, and resembled an allergic reaction with bronchospasm, promptly responding to bronchodilators with or without glucocorticoids. In one patient, however, it manifested subacutely as cough and dyspnoea, and was rated grade 4. Complete resolution of symptoms and CT-documented bilateral interstitial infiltrates was achieved by steroids in this patient also. Since this patient had experienced minor acute respiratory symptoms during the previous cycle, occurrence of this life-threatening condition might have been preventable. All other non-haematological toxicities (vomiting, diarrhoea, mucositis and hair loss) were of mild to moderate intensity and were recorded only in a minority of patients.

In conclusion, the results of our study indicate that vinorelbine, mitomycin C plus G-CSF has an excellent anti-tumour activity in advanced breast cancer. Therapeutic results, in fact, compare very favourably with the best results obtained with other standard regimens. An important additional advantage of this combination is its apparent non-cross-resistance with anthracyclines, as indicated both by objective responses in anthracycline-pretreated patients and anthracycline-induced remissions after progression to vinorelbine, mitomycin C plus G-CSF. Overall toxicity was moderate with myelosuppression being the dose-limiting side-effect. Severe non-haematological adverse reactions were relatively uncommon, and most of them, including constipation, local toxicity and (sub)acute lung toxicity, might be avoided by intensified individual concomitant medications, careful drug administration plus shortening of the infusion time (Hohneker, 1994), as well as clinical monitoring of all patients for acute respiratory symptoms during drug administration.

**Acknowledgement**
This study was supported in part by the Austrian Cancer Society, Section of Niederösterreich.

### References

ANDERSON JR, BERNSTEIN L AND PIKE MC. (1982). Approximate confidence intervals for probabilities of survival and quantiles in life-table analysis. *Biometrics*, **38**, 407-416.

AYASH LJ, ELIAS A, WHEELER C, REICH E, SCHWARTZ G, MAZANET R, TEPLER I, WARREN D, LYNCH C, GONIN R, SCHNIPPER L, FREI E AND ANTMAN K. (1994). Double dose-intensive chemotherapy with autologous marrow and peripheral-blood progenitor support for metastatic breast cancer: a feasibility study. *J. Clin. Oncol.*, **12**, 37-44.

BINET S, CHAINEAU E, FELLOUS A, LATASTE H, KRIKORIAN A, COUZINIER JP AND MEININGER V. (1990). Immunofluorescence study of the action of navelbine, vincristine and vinblastine on mitotic and axonal microtubules. *Int. J. Cancer*, **46**, 262-266.

BONADONNA G, VALLAGUSSA P, MOLITERNI A, ZAMBANETTI M AND BRAMBILLA C. (1995). Adjuvant cyclophosphamide, methotrexate and fluorouracil in node positive breast cancer: the results of 20 years follow-up. *N. Engl. J. Med.*, **14**, 332-341.

CANNOBIO L, BOCCARDO F, PASTORINI G, BREMA F, MARTINI C, RESASCO M AND SANTI L. (1989). Phase II study of navelbine in advanced breast cancer. *Semin. Oncol.*, **16**, 33-36.

CHEVALLIER B, FUMOLEAU P, KERBRAT P, DIERAS V, ROCHE H, KRAKOWSKI I, AZLI N, BAYSSAS M, LENTZ MA AND VAN GLABBEKEN M. (1995). Doxetaxel is a major cytotoxic drug for the treatment of advanced breast cancer: a phase II trial of the Clinical Screening Cooperative Group of the European Organization for Research and Treatment of Cancer. *J. Clin. Oncol.*, **13**, 314-322.

CLAVEL M AND CATIMEL G. (1993). Breast cancer: chemotherapy in the treatment of advanced disease. *Eur. J. Cancer*, **29A**, 598-604.

DEGARDIN M, BONNETERRE J, HECQUET B, PION JM, ADENIS A, HORNER D AND DEMAILLE A. (1994). Vinorelbine (navelbine) as a salvage treatment for advanced breast cancer. *Ann. Oncol.*, **5**, 423-426.

DEN HARTIG J. VERWEIJ AND PINEDO HM. (1995). Mitomycin C. In *Cancer Chemotherapy*, Pinedo HM and Chabner BA. (eds), Annual 7, pp. 83-90. Elsevier: Amsterdam.

FABI A, TONACHELLA R, SAVARESE A, CIRULLI S, TOMAO S, CONTE E AND COGNETTI F. (1995). A phase II trial of vinorelbine and thiotepa in metastatic breast cancer. *Ann. Oncol.*, **6**, 187-189.

FALKSON GF, GELMAN R, FALKSON CI, GLICK J AND HARRIS J. (1991). Factors predicting for response, time to treatment failure, and survival in women with metastatic breast cancer treated with DAVTH: a prospective Eastern Cooperative Oncology Group study. *J. Clin. Oncol.*, **9**, 2153-2161.

FUMOLEAU P, DELGADO FM, DELOZIER T, MONNIER A, GIL DELGADO MA, KERBRAT P, GARCIA-GIRALT E, KEILING R, NAMER M, CLOSON MT, GOUDIER MJ, CHOLLET P, LECOURT L AND MONTCUQUET P. (1994). Phase II trial of weekly intravenous vinorelbine in first-line advanced breast cancer chemotherapy. *J. Clin. Oncol.*, **11**, 1245-1252.

GARCIA-CONDE J, LLUCH A, CASADO A, GERVASIO H, DE OLIVEIRA C, DE PABLO JL, GOROSTIAGA J, GIRON GC, CERVANTES A, MARTINEZ A, PEZOUS N, DELGADO FM AND DIAZ RUBIO E. (1992). Phase II trial with navelbine in advanced breast cancer. *Breast Cancer Res. Treat.*, **23**, 143-145.

GASPARINI G, CAFFO O, BARNI S, FRONTINI L, TESTOLIN A, GUGLIELMI RB AND AMBROSINI G. (1994). Vinorelbine is an active antiproliferative agent in pretreated advanced breast cancer patients: a phase II study. *J. Clin. Oncol.*, **12**, 2094-2101.

HOHNEKER JA. (1994). A summary of vinorelbine (navelbine) safety data from North American clinical trials. *Semin. Oncol.*, **21** (suppl. 10), 42-47.

HOLMES FA, WALTERS RS, THERIAULT RL, FORMAN AD, NEWTON LK, RABER MN, BUZDAR AU, FRYE DK AND HORTOBAGYI GN. (1991). Phase II trial of taxol, an active drug in the treatment of metastatic breast cancer. *J. Natl Cancer Inst.*, **83**, 1797-1805.

HORTOBAGYI GN. (1993). Mitomycin C, its evolving role in the treatment of breast cancer. *Oncology*, **50** (suppl. 1), 1-8.

KORNEK GV, RADERER M, DEPISCH D, HAIDER K, FAZENY B, DITTRICH C AND SCHEITHAUER W. (1994). Amonafide as first-line chemotherapy for metastatic breast cancer. *Eur. J. Cancer*, **30A**, 398-400.

LUEDKE D, MCLAUGHLIN TT, DAUGHADAY C, DAUGHADAY C, LUEDKE S, HARRISON B, REED G AND ORLANDO M. (1985). Mitomycin C and vindesine associated pulmonary toxicity with variable clinical expression. *Cancer*, **55**, 542-545.

MANGENEY P, ANDRIAMIALISOA RZ AND LALLEHAND JY. (1979). 5′-Noranhydro-vinblastine. Prototype of a new class of vinblastine derivatives. *Tetrahydron*, **35**, 2175-2179.

MILLER AB, HOOGSTRATEN B AND STAQUET M. (1981). Reporting results of cancer treatment. *Cancer*, **147**, 207-214.

NOLE F, DE BRAUD F, DE PAS M, CASTAGNA L, COVELLI A AND AAPRO S. (1995). A phase I study of navelbine and fluorouracil plus folinic acid in patients with metastatic breast cancer (abstract 431). In *Proceedings of the 5th International Cancer Congress on Anticancer Chemotherapy*, Paris. 31 Jan-3 Feb.

POUILLART P, HOANG HT, BRUGERIE E AND LHERITER J. (1974). Sequential administration of two oncostatic drugs: studies of modalities for pharmacodynamic potentiation. *Biomedicine*, **21**, 471-479.

REICHMAN BS, SEIDMAN AD, CROWN JPA, HEELAN R, HAKES TB, LEBWOHL DE, GILEWSKI TA, SURBONE A, CURRIE V, HUDIS CA, YAO TJ, KLECKER R, JAMIS-DOW C, COLLINS J, QUINLIVAN S, BERKERY R, TOOMASI F, CANETTA R, ISHERMAN J, ARBUCK S AND NORTON J. (1993). Paclitaxel and recombinant human granulocyte colony stimulating factor as initial chemotherapy for metastatic breast cancer. *J. Clin. Oncol.*, **11**, 1943-1951.

ROMERO A, RABINOVICH MG, VALLEJO CT, PEREZ JE, RODRIGEZ R, CUEVAS MA, MACHIAVELLI M, LACAVA JA, LANGHI M, ROMERO ACUNA L, AMATO S, BARIERI R, SABATINI C AND LEONE BA. (1994). Vinorelbine as first-line chemotherapy for metastatic breast carcinoma. *J. Clin. Oncol.*, **12**, 336-341.

ROWINSKY EK, CAZENAVE LA AND DONEHOWER RC. (1990). Taxol: a novel investigational antimicrotubule agent. *J. Natl Cancer Inst.*, **82**, 1247-1259.

SCHEITHAUER W, DITTRICH C, KORNEK GV, HAIDER K, LINKESCH W, GISSLINGER H AND DEPISCH D. (1991). Phase II study of amonafide in advanced breast cancer. *Breast Cancer Res. Treat.*, **20**, 63-67.

SCHEITHAUER W, KORNEK GV, HAIDER K, KWASNY W, SCHENK T, PIRKER R AND DEPISCH D. (1993). Effective second-line chemotherapy for advanced breast cancer with navelbine and mitomycin C. *Breast Cancer Res. Treat.*, **26**, 49-53.

SORENSEN JB. (1995). Current position of vinorelbine in cancer chemotherapy. *Ann. Oncol.*, **6**, 105-107.

SPIELMANN M, DORVAL T, TURPIN F, ANTOINE E, JOUVE M, MAYLEVIN F, LACOMBE D, ROUSSE J, POUILLART P, TURSZ T AND MERLE S. (1994). Phase II trial of vinorelbine/doxorubicin as first-line therapy of advanced breast cancer. *J. Clin. Oncol.*, **12**, 1764-1770.

TOUSSAINT C, IZZO J, SPIELMANN M, MERLE S, MAY-LEVIN F, ARMAND JP, LACOMBE D, TURSZ T, SUNDERLAND M, CHABOT GG AND CVITKOVIC E. (1994). Phase I/II trial of continuous infusion vinorelbine for advanced breast cancer. *J. Clin. Oncol.*, **12**, 2102-2112.

VALAGUSSA P, BONADONNA G AND VERONESI A. (1978). Patterns of relapse and survival following radical mastectomy. *Cancer*, **41**, 1170-1172.